# Application of Image Processing Techniques in Molecular Imaging of Cancer

**DOI:** 10.1155/2017/2758749

**Published:** 2017-11-13

**Authors:** Bingsheng Huang, Guoyan Zheng, Ziyue Xu, Shengxiang Rao, Silun Wang

**Affiliations:** ^1^School of Biomedical Engineering, Health Science Centre, Shenzhen University, Shenzhen, China; ^2^Institute for Surgical Technology and Biomechanics, University of Bern, Bern, Switzerland; ^3^Center for Infectious Disease Imaging (CIDI), Radiology and Imaging Science Department, National Institutes of Health (NIH), Bethesda, MD 20892, USA; ^4^Department of Radiology, Zhongshan Hospital, Fudan University, Shanghai, China; ^5^Department of Radiology and Imaging Sciences, Emory University School of Medicine, Atlanta, GA, USA

Cancer is one of the leading causes of death all over the world. The research of cancer has always been a major focus in the medical imaging field. Modern imaging technology such as molecular imaging has already been shown to be useful in enhancing cancer management, including early detection, more accurate diagnosis, better treatment planning, and treatment monitoring in an early stage. Molecular imaging enables in vivo visualization and measurement of biological process at the cellular and molecular level. It allows not only localization of tumor lesions but also visualization of the expression and activity of specific molecules, which have great influence on tumor behavior and response to treatment. Many different modalities, such as MRI, SPECT, and PET, have been developed and used for noninvasive molecular imaging and have played a critical role in clinical oncology. However, due to large intersubject variability and various parameters in molecular images, it is generally infeasible to derive a single analytic method or simple equations that can describe the targets such as lesions and anatomies in all the images. Hence, in order to facilitate further the application of molecular imaging in clinical oncology, image processing techniques have been widely applied to the detection of cancer, characterization and segmentation of tumor lesions, planning of cancer treatment, evaluation of the effectiveness of treatment, prognostication of cancer, and so forth. Therefore, advanced image processing techniques have become a major focus in molecular imaging research, so that we can make better use of the rich information in the molecular image data.

The aim of this special issue is to provide a platform for high quality works on image processing and molecular imaging of cancer. Original papers and review articles focusing on the latest application of image processing techniques in multimodality cancer molecular imaging were submitted. The topics included pharmacokinetic modeling approaches, computer-aided detection/diagnosis of cancer, treatment evaluation and prognostication of cancer, segmentation/delineation of tumor lesions, correlation between molecular image data and other medical data of cancer from a medical perspective, advantages and limitations of existing and new imaging processing software/techniques, the importance of molecular image processing within the entire cycle of cancer patient management, and some other image processing techniques applied in cancer molecular imaging. We received a total of 16 submissions, and after two rounds of rigorous review, 5 papers were accepted for publications in this special issue.

In the paper “Head and Neck Cancer Tumor Segmentation Using Support Vector Machine in Dynamic Contrast-Enhanced MRI,” W. Deng et al. proposed an automatic method based on Support Vector Machine (SVM) and Dynamic Contrast-Enhanced Magnetic Resonance Imaging (DCE-MRI) to segment the tumor lesions of head and neck cancer (HNC). They calculated five curve features and two principal components of the normalized time-intensity curve (TIC) and trained three SVM classifiers. Compared to similar studies in literature, their method has achieved higher accuracy, and the average area overlap measure (AOM) with the testing dataset was 0.76 ± 0.08. This proposed method is of potential in the clinical practice for HNC.

In the paper “PET Imaging of FSHR Expression in Tumors with 68Ga-Labeled FSH1 Peptide,” D. Pan et al. developed ^68^Ga labeled FSH1 peptide for imaging of FSHR in cancers. ^68^Ga-NOTA-MAL-FSH1 was produced within 20 min and the radiochemical purity was greater than 95%. In vitro studies and MicroPET imaging were performed in PC-3 prostate tumor model. It showed that ^68^Ga -NOTA-MAL-FSH1 possessed FSHR binding affinities. The tracer was stable in human serum for at least 2 hours. MicroPET imaging revealed that the PC-3 xenografts were clearly visualized. FSHR binding specificity was also demonstrated by reduced tumor uptake of ^68^Ga-NOTA-MAL-FSH1 after coinjecting excess unlabeled FSH1 peptide. The favorable characters of ^68^Ga-NOTA-MAL-FSH1 such as convenient synthesis and specific tumor uptake warrant its further investigation for FSHR expression imaging.

In the paper “An Individually Optimized Protocol of Contrast Medium Injection in Enhanced CT Scan for Liver Imaging,” S.-T. Feng et al. investigated the effectiveness of a new individualized contrast medium injection protocol for enhanced liver CT scan. Patients who underwent plain and dual phase enhanced liver CT were randomly assigned to 2 groups, one with individualized contrast medium injection protocol and the other with standard contrast medium injection. The mean contrast medium dose was statistically lower with the individualized protocol. There were no significant differences in CT values and ΔHU (CT value difference between plain and enhanced CT) of liver parenchyma and tumor-liver contrast between two groups. Two independent radiologists were in substantial conformity in grading tumor conspicuity. The authors concluded that using the individually optimized injection protocol might reduce contrast medium dose without impacting on the imaging quality in enhanced liver CT.

In the paper “Dynamic Contrast-Enhanced Magnetic Resonance Imaging of Regional Nodal Metastasis in Nasopharyngeal Carcinoma: Correlation with Nodal Staging,” B. Huang et al. determined if the perfusion parameters by DCE-MRI of regional nodal metastasis were helpful in characterizing nodal status and to understand the relationship with those of primary tumor of nasopharyngeal carcinoma (NPC). 26 newly diagnosed patients with enlarged retropharyngeal/cervical lymph nodes suggestive of nodal disease were recruited and DCE-MRI was performed. Three quantitative parameters, *K*^trans^, *v*_e_, and *k*_ep_, were calculated for the largest node in each patient and analyzed. *K*^trans^ was significantly different among the patients of N stages. There was no significant correlation between the parameters in nodes and primary tumors. The authors concluded that DCE-MRI may play a distinct role in characterizing the metastatic cervical lymph nodes of NPC.

In the review article “Application of Deep Learning in Automated Analysis of Molecular Images in Cancer: A Survey,” Y. Xue et al. review the applications of deep learning in molecular imaging in terms of tumor lesion segmentation, tumor classification, and survival prediction. They also outline some future directions in which researchers may develop more powerful deep learning models for better performance in the applications in cancer molecular imaging.

